# Supplementary material for: Lsm12 is an NAADP receptor and a two-pore channel regulatory protein required for calcium mobilization from acidic organelles
Source: Nat Commun. 2021 Aug 6;12:4739. doi: 10.1038/s41467-021-24735-z (PMC8346516; doi:10.1038/s41467-021-24735-z)
Supplement: Supplementary file 1 — Supplementary Information [file 41467_2021_24735_MOESM1_ESM.pdf]

Supplementary Information

Lsm12 is an NAADP receptor and a two-pore channel regulatory protein required for calcium mobilization from acidic organelles

Jiyuan Zhang[1,3], Xin Guan[1,3], Kunal Shah[1], and Jiusheng Yan[1,2,*]

[1]Department of Anesthesiology & Perioperative Medicine, The University of Texas MD Anderson Cancer Center, Houston, Texas, USA

[2]Neuroscience and Biochemistry & Cell Biology Programs, The University of Texas MD Anderson Cancer Center UT Health Graduate School of Biomedical Sciences, Houston, Texas, USA

[3]These authors contributed equally to this work

*Correspondence and requests for materials should be addressed to J.Y. (jyan1@mdanderson.org).

Table 1. List of proteins as differential proteins between the test and control samples identified by quantitative mass spectrometric analysis from TPC1- and TPC2-expressing HEK293 cells.

**TPC1 interactome**					**TPC2 interactome**				
**UniProt ID**	**Mass**	**Score[1]**	**# peptides[2]**	**H/L ratio[3]**	**UniProt ID**	**Mass**	**Score[1]**	**# peptides[2]**	**H/L ratio[3]**
TPC1	94,147	9619	164	10.1	TPC2	85,243	6676	96	5.5
SYFM	52,357	159	2	15.4	FARP1	118,633	230	5	55.1
PRPS1	34,834	158	4	3.9	TIM50	39,646	153	5	4.5
MDHC	36,426	128	2	4.6	RT25	20,116	114	6	8.7
**LSM12**	21,701	126	2	1,586.3	RAD50	153,892	105	2	3.9
RT28	20,843	118	2	4.4	TDRD3	73,185	98	2	9.5
NEP1	26,720	114	2	4.1	BMP2K	129,172	96	2	3.2
**FHAD1**	161,904	106	6	8.3	**C19L1**	60,619	96	2	6.5
TRI26	62,166	77	4	8.3	**OBSCN**	868,484	90	2	16.4
PGRC1	21,671	76	2	18.8	LIN7A	25,997	84	2	278.7
**SH3R1**	93,129	74	8	29.1	ATPO	23,277	78	2	3.7
TOP3A	112,372	73	5	42.3	C2D1A	104,062	77	2	4.3
S30BP	33,870	72	2	2,084.3	PEF1	30,381	77	1	75.4
RT35	36,844	69	2	4.0	EPB41	97,017	73	3	7.9
**C19L1**	60,619	66	2	3.2	NOL12	24,663	67	3	30.4
ERP44	46,971	65	4	10.1	CCNL2	58,147	66	2	179.6
**OBSCN**	868,484	63	4	18,503.0	**LSM12**	21,701	66	4	6.8
CGBP1	18,820	63	3	43.9	MGME1	39,421	65	2	1,412.8
CLP1	47,646	62	4	97.3	ZO2	133,958	65	5	9.1
IMDH1	55,406	58	4	13.0	MIRO2	68,118	64	2	9.5
PRS7	48,634	56	2	4.3	**SH3R1**	93,129	63	7	50.4
PRG4	151,061	55	4	10,131.5	CPNE1	59,059	62	1	9.7
NB5R4	59,474	55	4	39.9	SUCB2	46,511	61	4	44.5
GAS8	56,356	52	2	16.1	ZN700	86,232	61	4	4,612.0
**RM20**	17,443	51	1	5,180.0	SSH1	115,511	56	3	9.6
RM21	22,815	49	4	323.8	PININ	81,628	54	4	1,803.3
**PRS6A**	49,204	49	2	68.6	DCAF7	38,926	53	1	22.2
KINH	109,685	46	2	6.4	CENPP	33,165	52	2	11.3
RTN4	129,931	46	1	4.9	HEXI2	32,419	51	1	269.6
AT1A2	112,265	46	4	4.0	**FHAD1**	161,904	48	2	8.6
NDUA5	13,459	45	1	38.0	CAND1	136,376	45	1	7.0
NOMO1	134,324	44	2	43.0	LANC1	45,283	45	2	7.4
ATP4A	114,119	44	4	4.4	WDR1	66,194	45	1	26.0
LAMP2	44,961	43	1	3.0	PUR2	107,767	43	2	78.6
FIZ1	51,996	42	2	12,550.0	CBX4	61,368	42	1	56.5
**TCEA3**	38,972	42	3	13.9	GTF2I	112,416	41	2	331.6
ZN644	149,565	41	4	23.7	LLPH	15,225	41	1	3.3
S10AB	11,740	41	1	9.1	**RM20**	17,443	41	1	35.1
FOLR3	27,638	40	1	42,100.0	**PRS6A**	49,204	40	1	235.8
BIG2	202,038	40	2	6.2	**TCEA3**	38,972	40	2	658.0
**NAADP interactome (TPC1-expressing cells)**					**NAADP interactome (TPC2-expressing cells)**				
**UniProt ID**	**Mass**	**Score[1]**	**# peptides[2]**	**H/L ratio[3]**	**UniProt ID**	**Mass**	**Score[1]**	**# peptides[2]**	**H/L ratio[3]**
**CGL**	44,508	439	9	5.6	GSHR	56,257	453	5	3.2
**KCC2D**	56,369	363	6	3.8	DDX1	82,432	397	5	4.3
DHB4	79,686	229	1	3.8	PAIRB	44,965	389	8	3.1
**BUB3**	37,155	209	10	4.6	HNRH2	49,264	330	6	3.1
**PTBP1**	57,221	163	7	3.0	KIFC1	73,748	325	7	5.4
SMRC2	132,879	145	5	3.5	**PTBP1**	57,221	267	10	4.0
LRC47	63,473	139	2	3.3	**BUB3**	37,155	266	14	4.7
**ODPA**	43,296	138	5	3.1	**ODPA**	43,296	238	5	3.3

LDHA	36,689	129	2	12.0
**LC7L3**	51,466	128	2	7.8
NCKP1	128,790	113	4	3.6
NUFP2	76,121	109	5	4.8
TBB3	50,433	109	2	12.2
WDR48	76,210	109	2	3.1
CAF1B	61,493	104	3	3.2
ILF2	43,062	103	2	3.6
**FBX22**	44,508	101	2	3.2
LONM	106,489	97	2	3.0
**MCM5**	82,286	97	6	3.4
PDIA4	72,932	97	3	3.6
HCFC1	208,732	92	2	3.2
**DRG1**	40,542	90	2	5.7
CPSF7	52,050	87	3	3.6
ACSL3	80,420	84	2	3.9
LMF2	79,698	79	1	4.9
TOE1	56,548	79	1	5.2
CBR4	25,301	74	1	4.0
**MCM3**	90,981	74	2	4.1
MPP6	61,117	74	5	3.7
OSGEP	36,427	74	1	5.9
HPRT	24,579	71	1	3.1
KHDR1	48,227	70	4	3.9
CPT1A	88,368	69	1	214.3
**SEPT2**	41,487	68	2	3.0
ANM1	42,462	67	2	3.8
EMC1	111,759	67	3	3.9
RPRD2	156,020	67	1	4.1
**CARF**	61,125	63	2	4.0
CYTSA	124,602	62	2	4.1
AAAS	59,574	61	1	4.4
RBBP5	59,153	60	1	3.6
**AATM**	47,518	59	3	3.0
RAI14	110,041	57	1	115.8
SEPT7	50,680	56	2	4.9
DMAP1	52,993	55	2	3.5
AL1B1	57,206	54	2	3.9
HS90A	84,660	54	2	3.2
**DDX47**	50,647	53	1	3.5
GNAS1	111,025	53	3	3.1
RT27	47,611	52	1	3.2
**CKAP2**	76,987	51	2	3.6
MFR1L	31,957	51	1	3.1
SYIM	113,792	50	1	4.4
CLAP2	141,064	48	1	10.7
VDAC1	30,773	46	1	6.3
GCDH	48,127	43	1	4.1
GLNA	42,064	42	1	8.6
HNRPQ	69,603	42	1	4.6
TBL1R	55,595	42	1	7.2
**LSM12**	21,701	41	1	3.0
SCLY	48,149	41	3	3.2
**SPF45**	44,962	41	3	4.3
PARN	73,451	40	1	5.4
KIF2C	81,313	189	6	5.4
**CGL**	44,508	184	9	5.6
HNRPL	64,133	179	13	3.4
IPYR2	37,920	170	3	3.9
HNRPF	45,672	168	3	3.2
C1TM	105,790	162	10	3.8
HMGCL	34,360	154	7	3.4
**MCM3**	90,981	134	3	3.2
**FBX22**	44,508	113	2	5.1
**LSM12**	21,701	110	2	3.3
**SEPT2**	41,487	109	1	3.3
P5CR1	33,361	103	2	5.3
WDR82	35,079	103	2	5.8
MCCB	61,333	93	2	4.1
SYFB	66,116	92	3	4.0
**DRG1**	40,542	91	2	6.0
MIC60	83,678	90	2	3.2
SRPRB	29,702	88	3	3.2
**MCM5**	82,286	87	5	3.4
GCP60	60,593	84	2	3.4
**DDX47**	50,647	82	1	3.9
DLDH	54,177	82	2	3.4
FAD1	65,266	77	4	3.6
STRAP	38,438	75	1	3.5
**AATM**	47,518	72	3	4.2
FND3A	131,852	70	2	4.6
**KCC2D**	56,369	70	6	4.0
TPX2	85,653	67	4	9.7
MTDC	37,895	66	2	27.6
**CKAP2**	76,987	65	3	4.8
DDX6	54,417	64	3	3.3
TCOF	152,106	61	3	3.1
KCC2A	54,088	60	4	4.6
**CARF**	61,125	58	2	3.9
RAB14	23,897	58	2	4.7
MAVS	56,528	56	1	4.5
TCPQ	59,621	56	1	3.2
BT2A1	59,633	55	2	4.2
**SPF45**	44,962	55	3	5.2
HXB9	28,059	54	1	5.1
SRC	59,835	54	1	5.7
PDIA1	57,116	53	1	4.8
TRM1L	81,747	53	1	3.2
**LC7L3**	51,466	51	1	3.6
EWS	68,478	48	2	17.3
ZC11A	89,131	48	2	5.9
HSP7E	54,794	46	1	3.4
SP1	80,693	46	1	7.6
SUCA	36,250	46	1	4.9
SH3G1	41,490	45	1	3.0
MRP	19,529	42	1	3.1
ADAS	72,912	40	2	3.7
NSDHL	41,900	40	2	18,851.0
SPB6	42,622	40	2	3.3

[1] Total mascot score for the protein.
[2] Number of matched H/L peptide pairs used for calculation of the protein's H/L ratio.
[3] Median of the heavy/light ratio of the protein.
Only proteins with H/L ratio of ≥3 and a mascot score ≥40 are included.
Protein names overlap between TPC1 and TPC2 interactomes and between TPC1- and TPC2-expressing NAADP interacteromes are highlighted in bold. Lsm12 is underlined as it is the only protein overlaps between TPC and NAADP interactomes.

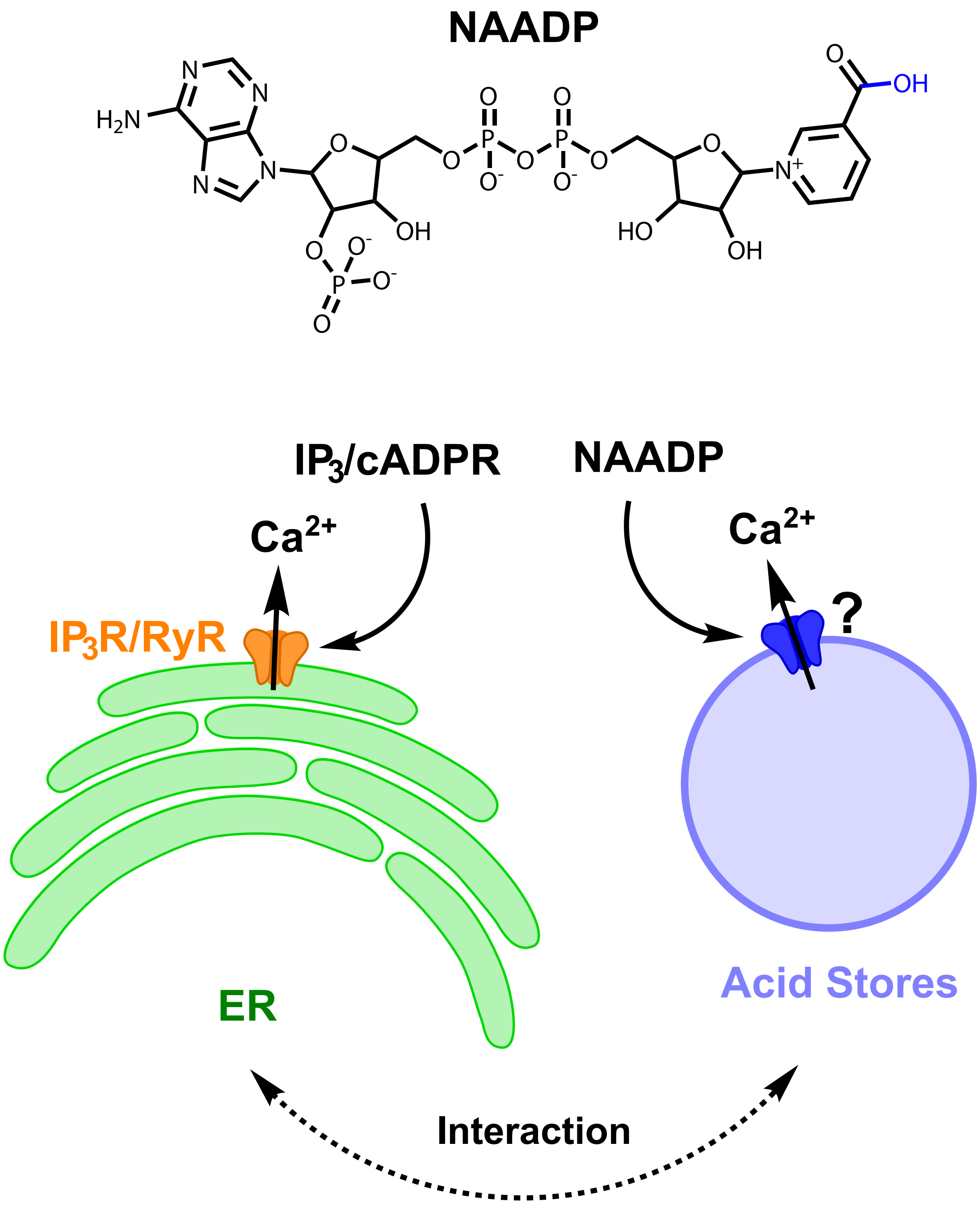

Figure 1. Chemical structure of NAADP and Ca^{2+}-mobilizing second messenger pathways. The -OH group, which differs from that ($-NH_2$) in NADP, is highlighted in blue in NAADP structure. A hypothetic TPC-containing multiprotein complex is proposed as the NAADP signaling complex responsible for NAADP-evoked Ca^{2+} release from acid stores.

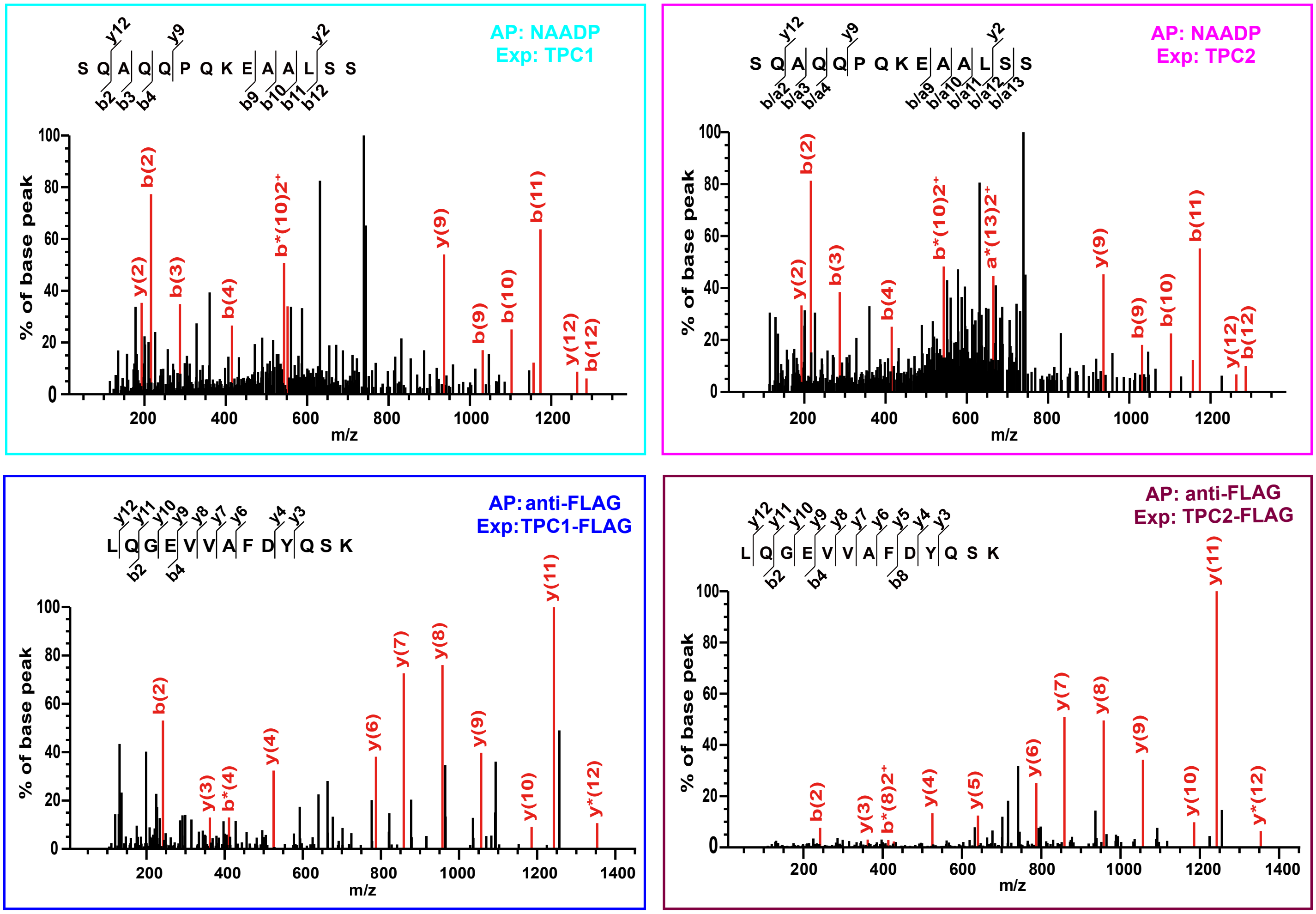

Figure 2. MS/MS spectra of Lsm12 peptides. MS/MS spectra of the peptide SQAQQPQKEAALS[194] and LQGEVVAFDYQSK[37] corresponding to those in Figure 1d. AP, affinity precipitation. Exp, expression.

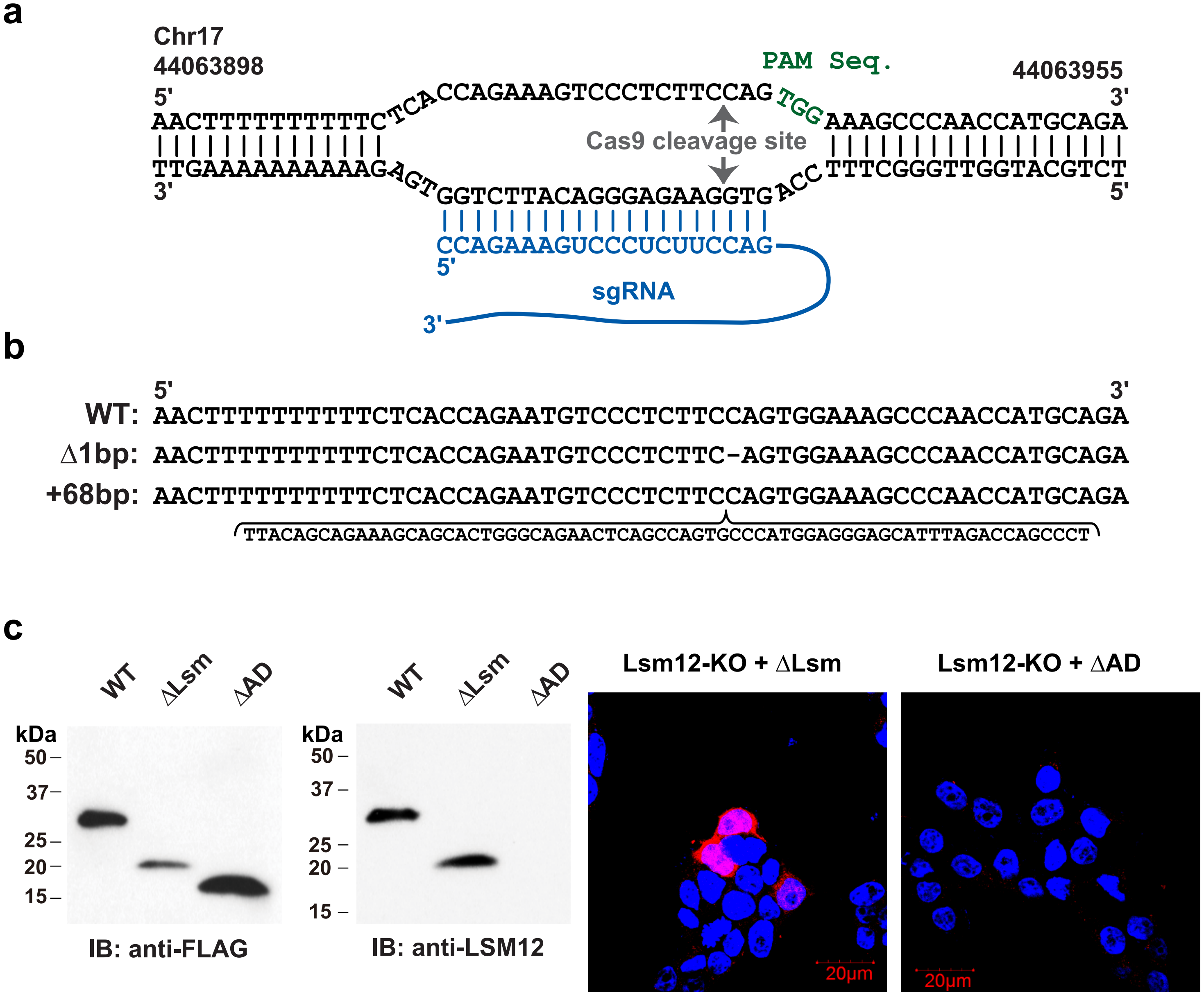

Figure 3. Generation and characterization of the Lsm12-KO line of HEK293 cells. a, Depiction of sgRNA-genomic DNA complex showing the targeted site of genomic editing in Lsm12 gene. **b**, Nucleotide sequences of Lsm12 genomic DNA in the sgRNA targeted region in HEK293 WT and Lsm12-KO cells. **c**, Immunoblot and immunofluorescence of Lsm12 by an anti-Lsm12 antibody of Lsm12-KO cells transiently expressing exogenous FLAG-tagged Lsm12-WT, -ΔLsm and -ΔAD mutants. Same amount of total protein of cell lysates was loaded for each lane in SDS-PAGE.

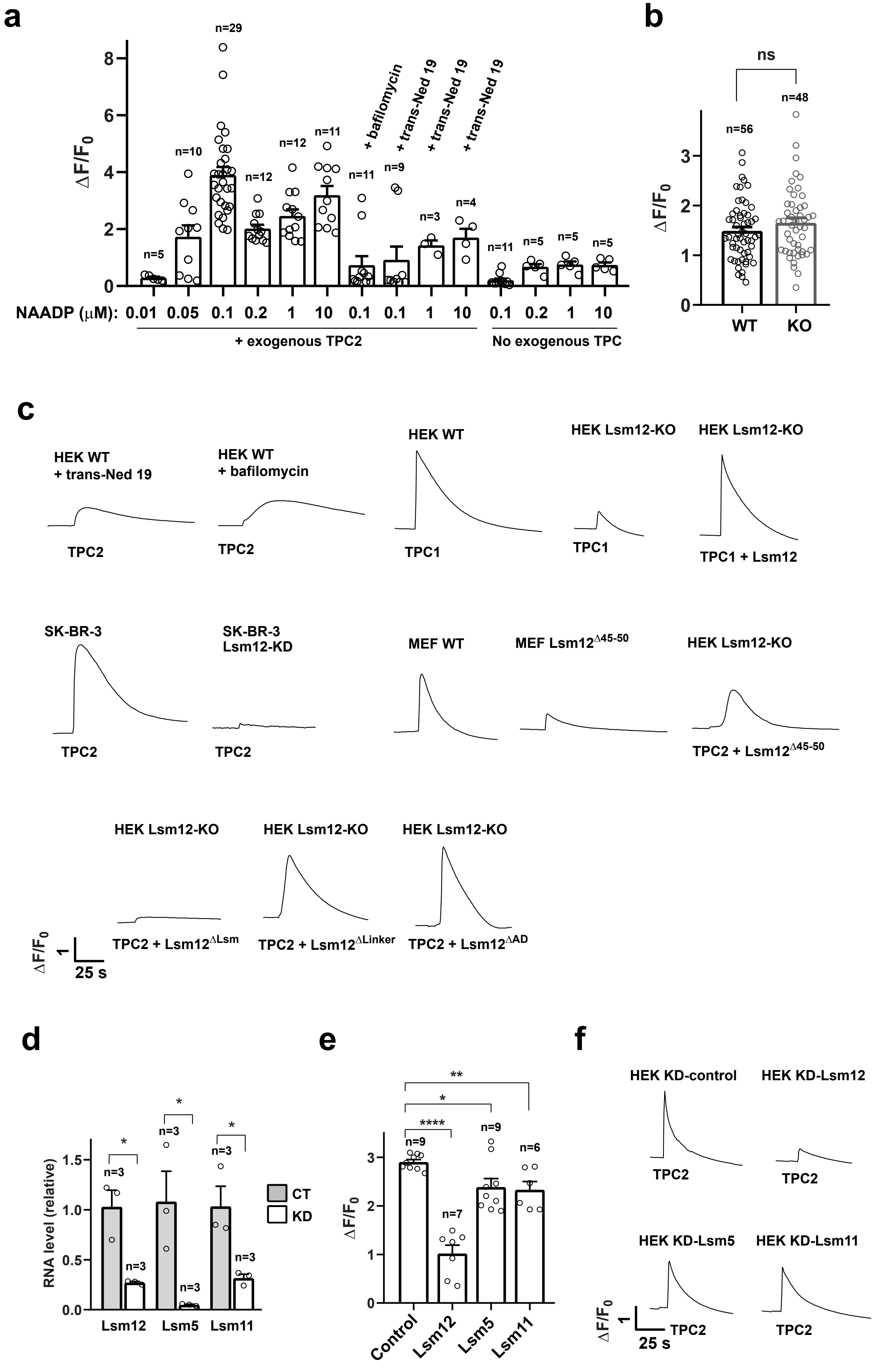
a
ΔF/F0
n=5
n=10
n=29
n=12
n=12
n=11
n=11
n=9
n=3
n=4
n=11
n=5
n=5
n=5
+ bafilomycin
+ trans-Ned 19
+ trans-Ned 19
+ trans-Ned 19
NAADP (μM): 0.01 0.05 0.1 0.2 1 10 0.1 0.1 1 10 0.1 0.2 1 10
+ exogenous TPC2
No exogenous TPC
b
ns
n=56
n=48
ΔF/F0
WT
KO
c
HEK WT + trans-Ned 19
TPC2
HEK WT + bafilomycin
TPC2
HEK WT
TPC1
HEK Lsm12-KO
TPC1
HEK Lsm12-KO
TPC1 + Lsm12
SK-BR-3
TPC2
SK-BR-3 Lsm12-KD
TPC2
MEF WT
MEF Lsm12Δ45-50
HEK Lsm12-KO
TPC2 + Lsm12Δ45-50
HEK Lsm12-KO
TPC2 + Lsm12ΔLsm
HEK Lsm12-KO
TPC2 + Lsm12ΔLinker
HEK Lsm12-KO
TPC2 + Lsm12ΔAD
ΔF/F0
1
25 s
d
RNA level (relative)
n=3
n=3
n=3
n=3
n=3
n=3
CT
KD
Lsm12
Lsm5
Lsm11
e
**
*
****
n=9
n=7
n=9
n=6
ΔF/F0
Control
Lsm12
Lsm5
Lsm11
f
HEK KD-control
TPC2
HEK KD-Lsm12
TPC2
HEK KD-Lsm5
TPC2
HEK KD-Lsm11
TPC2
ΔF/F0
1
25 s

Figure 4. NAADP-evoked Ca^{2+} changes in HEK293 cells under different conditions. a, The effect of different NAADP concentrations in injection pipette solution and different cell treatments on NAADP microinjection-induced change in the Ca^{2+} indicator fluorescence of HEK293 WT cells. Bafilomycin A1 at 1 µM or trans-Ned 19 at 10 µM was added in cell growth medium for 45- 60 min before Ca^{2+} imaging. **b**, Extracellular 1 µM ATP treatment–induced changes in intracellular Ca^{2+} were similar in WT and Lsm12-KO cells expressing exogenous TPC2. **c**, Time course of NAADP-induced change in fluorescence of Ca^{2+} indicator. The cells and treatment were indicated on top and the transfected plasmids (TPC or Lsm12) were shown at the bottom. **d**, RT-PCR shows knockdown of Lsm12, Lsm5 or Lsm11 expression by siRNA in HEK293 cells. Repeats were done on the same RNA samples. **e**, The effects of Lsm protein siRNA on NAADP-evoked change in the Ca^{2+} indicator fluorescence of HEK293 cells expressing exogenous TPC2. CT, control. KD, knockdown. **f**, Time course of NAADP-induced change in fluorescence of Ca^{2+} indicator after knockdown (KD) treatment. Data are presented as mean value +/-SEM. Unpaired Student's t-test (two tailed) was used to calculate *p* values. ns, *, ** and **** are for *p* values >0.05, ≤0.05, ≤0.01 and 0.0001, respectively.

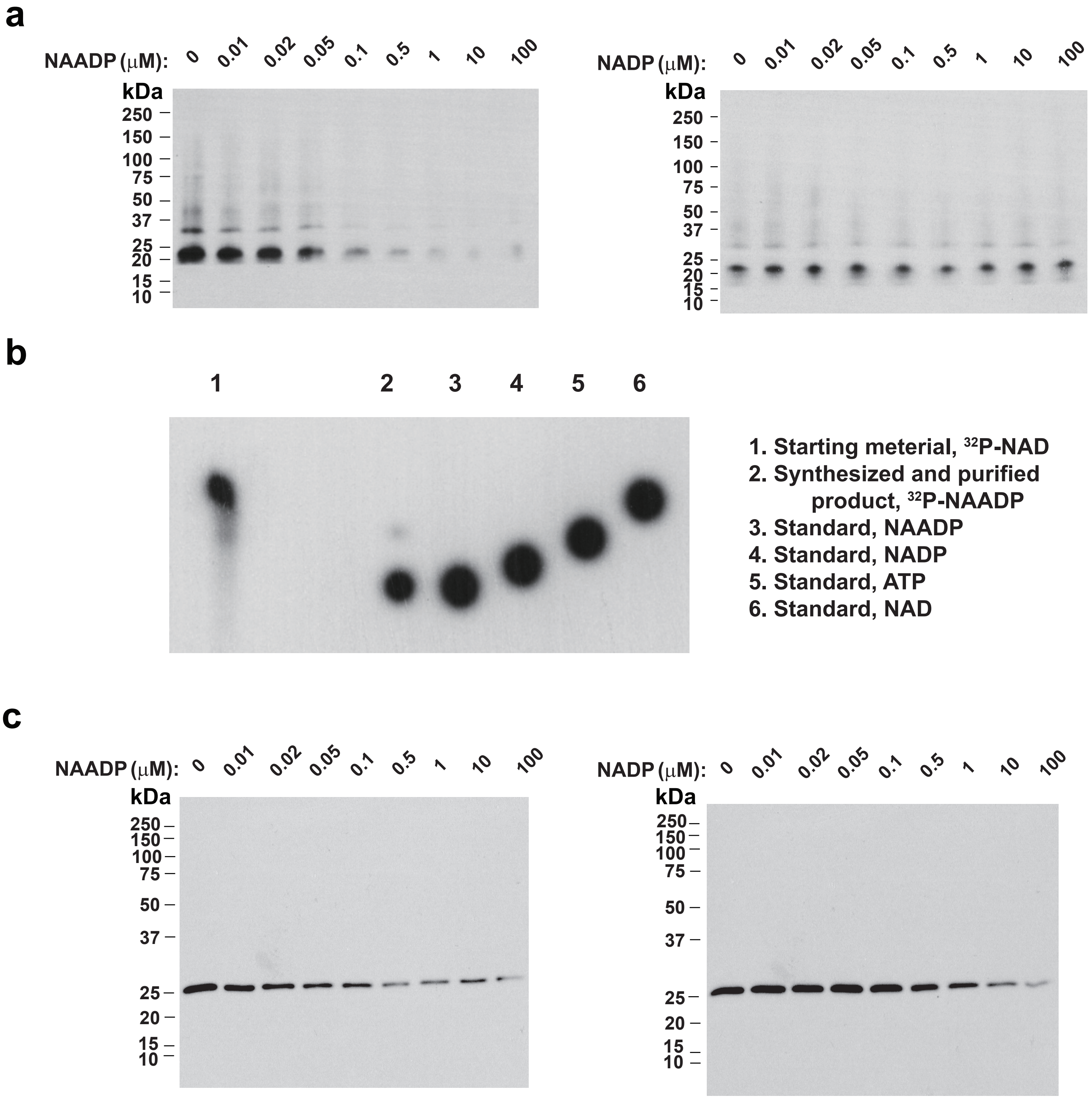

Figure 5. Competition binding assay of NAADP binding to hLsm12-His$_{E.coli}$ and TPC2-expressing HEK293 cell membranes. a, Immunoblot of hLsm12-His$_{E.coli}$ antibody pulled down by immobilized NAADP in the absence or presence of various concentrations of free NAADP and NADP. **b**, Thin-layer chromatography (TLC) of synthesized and purified ^{32}P-NAADP. The image was obtained by autoradiography. The standard NAADP, NADP, ATP, and NAD were visualized under a UV lamp on TLC plate first and then spotted with ^{32}P. **c**, Immunoblot of endogenous Lsm12 in HEK293 cells pulled down by immobilized NAADP in the absence or presence of various concentrations of free NAADP and NADP. Immunoblot was done with anti-Lsm12 antibody.

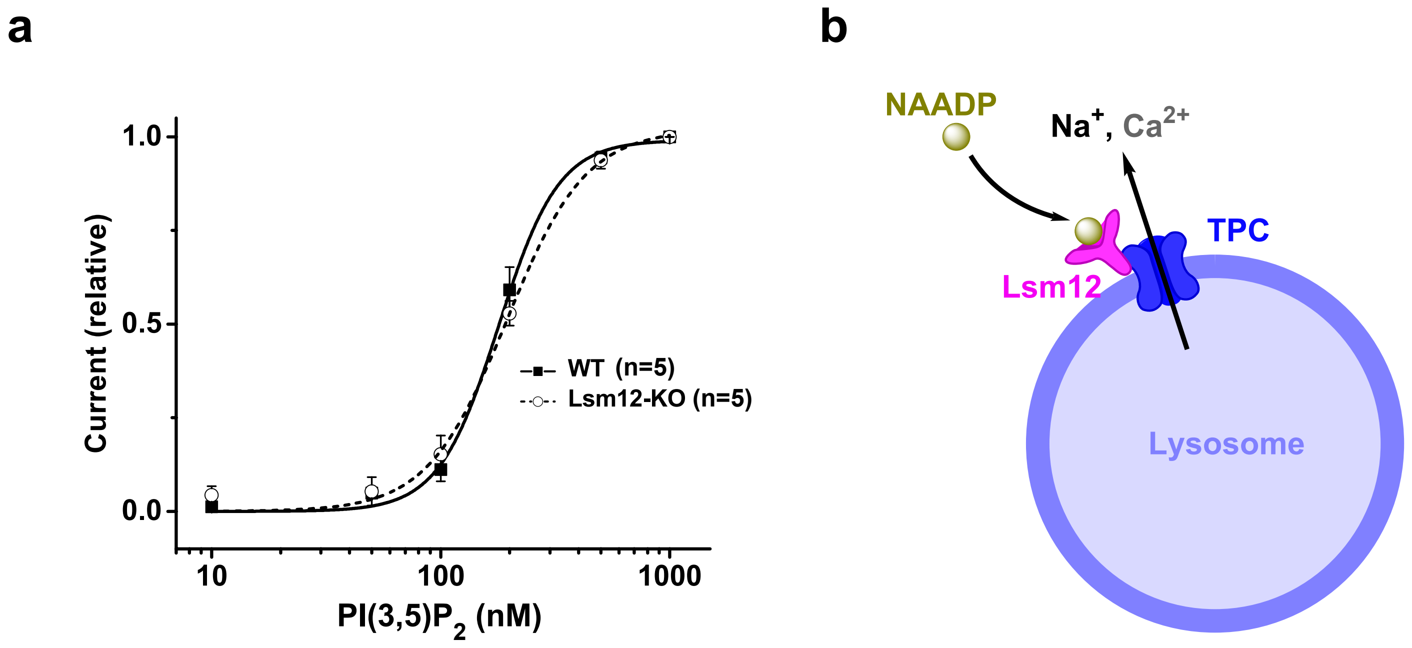

Figure 6. Dose response of endolysosomal TPC2 to PI(3,5)P_2 and proposed model of Lsm12 in NAADP signaling. a, Does response of endolysosomal TPC2 to PI(3,5)P_2 obtained by whole lysosome (enlarged) patch-clamp recording. **b**, Proposed model of Lsm12 in NAADP signaling. Data are presented as mean value +/-SEM.
