# Supplementary material for: Lsm12 is an NAADP receptor and a two-pore channel regulatory protein required for calcium mobilization from acidic organelles
Source: Nat Commun. 2021 Aug 6;12:4739. doi: 10.1038/s41467-021-24735-z (PMC8346516; doi:10.1038/s41467-021-24735-z)
Supplement: Supplementary file 3 — Reporting Summary [file 41467_2021_24735_MOESM3_ESM.pdf]

nature research

Corresponding author(s): Jiusheng Yan

Last updated by author(s): 06/26/2021

Reporting Summary

Nature Research wishes to improve the reproducibility of the work that we publish. This form provides structure for consistency and transparency in reporting. For further information on Nature Research policies, see our Editorial Policies and the Editorial Policy Checklist.

Statistics

For all statistical analyses, confirm that the following items are present in the figure legend, table legend, main text, or Methods section.

n/a	Confirmed	
☐	☒	The exact sample size (n) for each experimental group/condition, given as a discrete number and unit of measurement
☐	☒	A statement on whether measurements were taken from distinct samples or whether the same sample was measured repeatedly
☐	☒	The statistical test(s) used AND whether they are one- or two-sided<br>*Only common tests should be described solely by name; describe more complex techniques in the Methods section.*
☒	☐	A description of all covariates tested
☐	☒	A description of any assumptions or corrections, such as tests of normality and adjustment for multiple comparisons
☐	☒	A full description of the statistical parameters including central tendency (e.g. means) or other basic estimates (e.g. regression coefficient) AND variation (e.g. standard deviation) or associated estimates of uncertainty (e.g. confidence intervals)
☐	☒	For null hypothesis testing, the test statistic (e.g. F, t, r) with confidence intervals, effect sizes, degrees of freedom and P value noted<br>*Give P values as exact values whenever suitable.*
☒	☐	For Bayesian analysis, information on the choice of priors and Markov chain Monte Carlo settings
☒	☐	For hierarchical and complex designs, identification of the appropriate level for tests and full reporting of outcomes
☒	☐	Estimates of effect sizes (e.g. Cohen's d, Pearson's r), indicating how they were calculated

Our web collection on statistics for biologists contains articles on many of the points above.

Software and code

Policy information about availability of computer code

Data collection	ZEN Blue 2 software containing Physiology module (Carl Zeiss), pCLAMP 10 (Axon Instruments)
Data analysis	ImageJ (1.52p), Igor Pro (5), OriginLab (2015, 2017), GraphPad Prism (8.0.0), Mascot (v2.4), Mascot Distiller (v2.6)

For manuscripts utilizing custom algorithms or software that are central to the research but not yet described in published literature, software must be made available to editors and reviewers. We strongly encourage code deposition in a community repository (e.g. GitHub). See the Nature Research guidelines for submitting code & software for further information.

Data

Policy information about availability of data

All manuscripts must include a data availability statement. This statement should provide the following information, where applicable:

- Accession codes, unique identifiers, or web links for publicly available datasets
- A list of figures that have associated raw data
- A description of any restrictions on data availability

The raw LC-MS/MS data generated in this study have been deposited in the MassIVE database (https://massive.ucsd.edu) under accession codes MSV000087415, MSV000087416, MSV000087417, and MSV000087418. The remaining data are available within the Article, Supplementary information or available from the authors upon request. Source data are provided with this paper.

Field-specific reporting

Please select the one below that is the best fit for your research. If you are not sure, read the appropriate sections before making your selection.

☒ Life sciences ☐ Behavioural & social sciences ☐ Ecological, evolutionary & environmental sciences

For a reference copy of the document with all sections, see nature.com/documents/nr-reporting-summary-flat.pdf

Life sciences study design

All studies must disclose on these points even when the disclosure is negative.

Sample size	For quantitative analysis of the difference, a sample size that was large enough to produce significant difference (p< 0.05 in t-test) between groups was chosen. For qualitative analysis, 2 or more independent experiments were usually done and in some cases no repeat was done because the result was fully negative and/o clear and/or fully supported by other data.
Data exclusions	No data was excluded unless there was known issue of the experiment, specifically the whole set of data from 1 independent experiment of Fig. 3a was not used because of sample loading problem for SDS-PAGE.
Replication	For quantitative analysis, a minimum of 3 repeats were done. For qualitative analysis, most experiments were done 2 or more times at the same or similar conditions. Replication was all successful.
Randomization	For calcium imaging and whole cell recording, cells qualified for analysis were randomly chosen but with similar expression of fluorescent indicator protein of transfection. For cell imaging, view field was chosen either randomly or to be representative.
Blinding	Blinding is not relevant to this study because all data were collected by instruments and not biased by the operators.

Reporting for specific materials, systems and methods

We require information from authors about some types of materials, experimental systems and methods used in many studies. Here, indicate whether each material, system or method listed is relevant to your study. If you are not sure if a list item applies to your research, read the appropriate section before selecting a response.

Materials & experimental systems

n/a	Involved in the study	
☐	☒	Antibodies
☐	☒	Eukaryotic cell lines
☒	☐	Palaeontology and archaeology
☐	☒	Animals and other organisms
☒	☐	Human research participants
☒	☐	Clinical data
☒	☐	Dual use research of concern

Methods

n/a	Involved in the study	
☒	☐	ChIP-seq
☒	☐	Flow cytometry
☒	☐	MRI-based neuroimaging

Antibodies

Antibodies used	Rabbit polyclonal anti-FLAG antibody (Cat# F7425 from Sigma-Aldrich), mouse anti-V5 (clone V5-10) agarose affinity gel (Cat# A7345 from Millipore), mouse monoclonal anti-FLAG M2 antibody (Cat# F3165 from Sigma-Aldrich), mouse monoclonal anti-V5 antibody (Cat# R96125 from Invitrogen), mouse monoclonal anti-V5 (Cat# SAB2702199 from Sigma-Aldrich), and rabbit monoclonal anti-LSM12 antibody (Cat# EPR12282 from Abcam).
Validation	All antibodies were validated in lab by using cells expressing the antigen and lacking the antigen. For western blot and IP, a clear presence and absence of the western blot band at the predicted molecular weight was used to validate the antibody. For immunofluorescence, the presence and absence of the signal in the immunostained cells expressing or lacking the antigen were used to validate the antibody. For anti-Lsm12 antibody, only human Lsm12 was tested for validation.

Eukaryotic cell lines

Policy information about cell lines

Cell line source(s)	Commercial sources of HEK293 (293H, Cat# 11631017) and SK-BR-3 cell lines (Cat# HTB-30 from ATCC) ware used.
Authentication	Cells were frequently regrown from stocks and no further authentication was performed.

Mycoplasma contamination	Anti-mycoplasma reagent was routinely included in the cell culture medium for HEK293 cells and the test for mycoplasma was negative. No treatment and test were done for SK-BR-3 cells.
Commonly misidentified lines (See ICLAC register)	No commonly misidentified cell lines were used.

Animals and other organisms

Policy information about studies involving animals; ARRIVE guidelines recommended for reporting animal research

Laboratory animals	C57BL/6 mice. E12.5 - E13.5 mouse embryos of both sexes were used for isolation of mouse embryonic fibroblasts.
Wild animals	No wild animal was used.
Field-collected samples	No field-collected sample was used.
Ethics oversight	All animal experiments were carried out according to protocols and guidelines approved by the Institutional Animal Care and Use Committee of The University of Texas MD Anderson Cancer Center.

Note that full information on the approval of the study protocol must also be provided in the manuscript.
